# CovaDel: a blockchain-enabled secure and QoS-aware drone delivery framework for COVID-like pandemics

**DOI:** 10.1007/s00607-022-01064-7

**Published:** 2022-03-14

**Authors:** Maninderpal Singh, Gagangeet Singh Aujla, Rasmeet Singh Bali, Ranbir Singh Batth, Amritpal Singh, Sahil Vashisht, Anish Jindal

**Affiliations:** 1 Chandigarh University, Mohali, India; 2 Durham University, Durham, UK; 3 Lovely Professional University, Phagwara, India; 4 Chitkara University Institute of Engineering and Technology, Chitkara University, Chandigarh, India; 5 Chandigarh Group of Colleges, Jhanjeri, India

**Keywords:** Blockchain, Firefly optimisation, Quality of service, Drone delivery, Security, 94A62, 94A60, 93A15, 90B22

## Abstract

With increase in the use of contactless deliveries during the times such as COVID-19 pandemic, the emphasis was to minimize the human presence to reduce the spread of virus. In this regard, drones are one promising alternative to be used as delivery agents. However, security and Quality of Service (QoS) are major concerns while making use of drones for deliveries. In order to secure the drone communication system, we propose, CovaDel: a blockchain-based scheme to secure data transactions for the drone delivery use case that works in a phased manner. The proposed scheme make use of decoupled blockchain architecture to overcome the limited resources capabilities of drones to perform blockchain-based computations. Further, to ensure the QoS adherence, we also propose a QoS-aware communication approach that handles collisions and congestion on the basis of firefly algorithm’s attractiveness parameter (light intensity) received by the drones. Results obtained using a simulated environment verify the efficacy of the proposed scheme on the basis of gas consumed, transaction time, average network throughput, and delay.

## Introduction

The transportation sector has helped the common life during the pandemic as it brought essential services and goods to the people. With the widespread emphasis of contactless deliveries of goods to the consumers, drones have become one potential solution for providing essential goods and services to the people. Many successful drone-based delivery services and systems are already in work such as, F-Drones, 7-Eleven, Swoop Aero [1], Zipline [2], Matternet, USPS HorseFly to name a few [3]. Drones offer many benefits to act as the promising delivery agents in COVID-like or disaster-hit scenarios, where the reach of humans is limited. These include their optimized designs, good coverage of area, low maintenance cost, and automated control with very less human involvement. In a typical drone delivery use-case, many entities are involved such as buyers, sellers, service providers, drones and their logistic planning. When all these different entities communicate with one another, it is essential to ensure prompt and secure information to the participating entities and deliver products at the right place in order to gain their trust through smooth operations and transactions. However, many security concerns (like, confidentiality, data manipulation, and authentication) and susceptibility of drones to cyber-attacks hinder their widespread rollout for delivering goods and general trust over their use [1]. Therefore, there is an urgent requirement of a streamlined system which can alleviate the security concerns and provide a secure mode of communication and trust between various involved entities.

In this regard, different researchers have tried to address these security concerns (like, confidentiality, data manipulation, event chronology, and authentication) in-part for different scenarios involving drones. For example, [4] proposed a framework to secure the Internet of Drone (IoD) ecosystem by protecting the drones by using multilevel and multi domain strategies. However, this often involves the use of heavy cryptographic computations which should be avoided in drone-based environment to save resources as well as provide speedy operations [5]. Although many research works have focused on the drones-based delivery services, there is still a major research gap concerning security in such systems for critical scenarios. One of the ways to address the security concerns is to use the blockchain technique which is based on a distributed ledger technology. The premise behind the use of blockchain is that the *blocks* in the blockchain are indestructible. It enables addition of data in the form of transactions and periodically update the transactions from various participating entities into blocks [6]. Overall, a block can be defined as the container of information in the form of chronological transitions thereby handling the issues like confidentiality and event chronology. In blockchain, every block has a header part and a trailer part. The header constitutes necessary control and administrative information required for ensuring consistency and verifiability of recorded transactions [7]. The trailer has actual transactions in it. To weave the blocks together, hashing operation is carried out to compute a fixed length output for any given input. The advantage of hashing is that for the same input, the output will be same, and even a minimal change in the input leads to a different output to a large extent.

Even though blockchain is an effective solution to deal with the security related issues, the computations involved in blockchain-based solutions can be computationally expensive for the (computation limited) drones. Blockchain has been found as a suitable solution for the drone delivery or other drone operations as pointed by many studies [8, 9], but very little exploration has been done for the adaptability of blockchain in the context of disaster or pandemic like situations. One of the key reasons for this little exploration is related to the resource constraints associated with the drones. If the complete blockchain including data (trailer) and header is stored on drone, the storage requirements of drones become very high over whelming the drone resources. The structure of conventional blockchain is appropriate for all entities, however, it has certain limitations when adopted in the drones scenario. Motivated from the work in [10], a derivative of blockchain can be used in the drone delivery scenario. In the derived blockchain, the data and header parts can be decoupled from the blockchain to keep the storage requirement on drones to the minimum. The blockchain will contain only the header part of different blocks. Moreover, each drone can have its own block which is appendable in nature. This way every time new data is added to the block of the drone or existing is updated the resulting hash of the block data changes. The hash of the data of drones is embedded into the blockchain of headers. A complete blockchain and derived blockchain can be maintained with the service provider (or drone station) including blocks for all drones but the drones store only their own block. Therefore, in our proposed approach - named as CovaDel, we make use of decoupled blockchain architecture to reduce the computational burden on drones.

When we have large number of resources, optimal utilization becomes essential to have the desired output of the system. So, if a drone-based delivery system is to be scaled commercially it must be designed keeping the optimal resource allocation and utilization in mind. Hence, to use drones for the purpose of delivery a optimized drone scheduler is needed [11, 12]. Specifically, in the case of drones, there are several attributes (status of physical resources, energy levels, and operational capabilities of hardware resources, etc) that must be taken into account before scheduling it for a mission or delivery. For example, if a drone is scheduled for delivery and during its flight any significant incident or delay occurs, then it may have to be divert its route or delivery plans. In such a case, it must have significant resources (like energy level) to sustain its flight considering the diversion or delay. If this is not considered by the scheduler then it may end up in delivery failure or any other untoward accident. Thus, effective scheduling mechanisms work as an enabler for ensuring that the quality of service (QoS) and quality of experience (QoE) parameters are met to make the system adaptable and worthy of deployment. In CovaDel, the scheduler considers all the above highlighted constraints before selecting a drone for delivery. To aid this process, a drone indexing approach is adopted that categorizes and index the drones based on its computational capabilities and physical resource status.

Moreover, the foundation of drone-based system relies primarily on the prompt communication between the involved entities and in this regard, some of the related communication concerns like, network congestion and collisions can have damaging impact on the overall QoS and QoE at the large. The density of the drones in a given area have big impact on the fluctuations of the bandwidth availability. This also impacts the on board resource utilization attributed to handling the network congestion [13]. Further, as the drones use wireless networks that uses shared channels with other coexisting networks like terrestrial and cellular networks resulting into interference, hence leading to collision of data packets. The corrupted data needs to be re-transmitted which is not always feasible due to the real time nature of the environment in which drones operate. Thus, because of this the drone delivery mechanisms should be robust enough to deal with the communication-based issues such as collisions and network congestion. In order to address this, CovaDel proposes a QoS-aware communication amongst the drones on the basis of firefly algorithm’s attractiveness parameter. Firefly algorithm is an effective optimization technique that can be applied to swarms of drones [14]. The key parameters for the firefly are attractiveness, randomization and absorption that is influenced from tropical firefly behaviour.

### Research approach and contributions

**Fig. 1:**
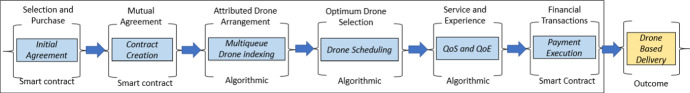
Schematic diagram for the research approach

CovaDel is focused on the delivery of essential goods (or medicines) using drones in pandemic or disaster-like scenarios when the physical moment is restricted (or limited). This article is the extension of our previous work [15] where we have provided the preliminary results and information on the use of blockchain for COVID-like scenarios. The overview of the research approach adopted is presented in the form of a schematic diagram depicted in Fig. 1.

In CovaDel, the block structure used for drone-to-drone, drone-to-ground station communication, and data storage differs from the traditional blockchain approach (Sect. 4). The approach workflow is based on different phases responsible for the functioning of CovaDel. For each phase, a self executing code, i.e., smart contracts, is designed for the interaction between the user and the buyer for placing an order (details given in Sect. 5.1). Furthermore, smart contracts are designed for ensuring the delivery of the items and payment processing between the involved entities (Sects. 5.2 and 5.3) respectively. Drones have different attributes in terms of their flight capabilities, so selecting the right drone for the right delivery is an essential job at hand which is achieved through a multi-queue drone indexing phase (Sect. 5.4). The scheduling of drones is discussed in Sect. 5.5. A good experience is the key for the popularity of any system among masses, hence a QoE enabling mechanism which is possible by ensuring the QoS for the drone communication becomes essential. Thus, a QoS-aware communication approach that handles collisions and congestion situation effectively is proposed in Sect. 6. Some of the significant contributions in this work are listed below.

- A systematic architecture for drone delivery use case has been presented wherein blockchain has been used for secure data transactions. A decoupled blockchain has been used to overcome the challenge of limited resources capabilities of drones concerning the heavy computational perspective of blockchain process.
- An end-to-end drone delivery mechanism using blockchain and multi-level queuing is designed considering different categories of drones based on their capabilities (such as weight, speed, fitness, battery, etc.).
- A QoS-aware communication approach that handles collisions and congestion is proposed on the basis of light intensity received by the drones.

## Related work

The existing literature in this field is presented in this section driven in two research directions focusing on (1) research aspects, and (2) drone delivery use cases.

### Research aspects in existing works

In [16], the authors evaluated a drone delivery case study related to healthcare field. The authors have studied various drone service providers, load capacities of various drones, and their suitability to the drone-based delivery services. Work by the authors in [17] includes the study of the environmental impact of using drone-based delivery services in comparison to motorcycle-based delivery of goods. The study clearly indicated the drone-based delivery holds superior to other means of goods delivery in terms of environmental impact. In [18], the authors have undertaken the study to provide an optimised delivery route planning using mobile ground station for the drones. The trajectory of the ground station is computed in a way that the ground station movement is minimum and the drones are capable of optimize their operations to maximize their coverage area. Kim et al. [19] suggested the use of mixed integer linear programming (MILP) model for planning drone activity to increase the count of drone-based parcel delivery that would take place on building rooftops. However, the major drawback of all these studies is that none of these works considered the security and privacy in drones.

In [20], the authors concluded that the any type of attack in drones can be fatal as it can stop its rotors mid air. Another similar work by authors in [21] on generic drone framework was presented along with its security assessment and resilience for security attacks like battery depletion, eavesdropping, jamming, replay and fabrication. In [22], the authors have presented a complete study of security aspects of IoT and related areas along with security mechanism to deal with the security concerns. All of these studies emphasize on the consideration of security flaws in drones, however, none of the above proposals considered a scenario related to product delivery or emergency delivery using drones. Since the delivery of commercial or non-commercial goods involve personal data or sensitive information related to the customers, it becomes essential to secure these transactions and maintain the integrity of the data. In view of these facts, the researchers in [1] have presented a framework for drone-based delivery of goods. The authors made use of cryptography to ensure confidentiality, authentication and non-repudiation. However, it seems that blockchain is better suited technology that can handle the various challenges concerning drone delivery. On one hand, blockchain can handle the transactions in drone delivery in a secure and tamper proof manner, while, on the other hand, we can utilize the smart contracts to verify and validate the authenticity of the transactions and flag out any potential violations.

Keeping in view of these facts, the authors in [23] presented blockchain, as a distributed ledger, which uses cryptographic methods to secure the shared data. It can also be used to ensure the accuracy of the data stored, as well as to improve the reliability and accountability of unmanned areal vehicles (UAVs). In another work [8], a framework for drone-based delivery empowered by blockchain was proposed to ensure system security. The proposed models uses practical Byzantine fault tolerance consensus mechanism for ensuring the consistence of the blockchain. The system was analysed against various security attacks. Another mechanism for validating the vendor packages was also presented in this work. In [9], the authors described the use of a blockchain and drone combination for delivery in countering COVID-19 to track, identify, handle, and regulate public health emergencies.

### Drone delivery – industrial/commercial use cases

Drone delivery is a massive technology which has proven itself to be an essential part of futuristic transportation systems. Drones helps to redefine the conventional logistic industry, which results in saving of crucial operational time, resources and the cost. This section provides information about the various drone delivery applications categorized into three domains discussed below.

- *Essentials and medicine* Contactless deliveries are booming during the pandemic time and drones are playing an important role in connecting consumer and the service providers by catering to such demands. Drones have become the first choice for delivering the essential goods such as medicine [24], first aid and sensitization during the lockdown time as well as in disaster-hit areas [25]. Drones even help to deliver the transplanted organs in time bound manner which can save one’s life [26].
- *e-commerce* The delivery of parcels and goods with the help drone is popular these days. The logistics companies such as DHL [27], Anavia [28] and Amazon [29], utilizes the drones for automated delivery of their products to the consumers. Some use-cases such as [30] exploits the use of drones in the marine deliveries of goods and containers to-and-from vessel to consumers locations and offshore. It is believed that this process helps to reduce the carbon footprints. This is derived due the huge demand of faster, cheaper and ideally green forms of deliveries.
- *Grocery and food delivery* 7 Eleven utilizes the drones for the delivery of grocery items to the customers [31]. To avail shop-to-home delivery, the customers register themselves on the site by providing the their home GPS coordinates for accurate delivery. Dominos, the famous pizza company, also focus on the use of drones to deliver hot pizzas in time bound delivery to its customers [32]. Moreover, a tacocopter (renamed from drone) to deliver tacos in San Fanscisco is designed by star simpson [33].

Table 1 show different drone delivery scenarios adopted by various organizations.

**Table 1:** Comparison of drone delivery cases

Case	Category	Delivery purpose	No. of drone	Payload
[24]	Essentials	Medicine	Single	Upto 10 kg
[26]	Essentials	Transplanted organs	Single	NA
[27]	e-commerce	Parcel	Single	Upto 2 kg
[28]	e-commerce	Parcel	Single	Upto 65 Kg
[29]	e-commerce	Goods	Single	NA
[30]	e-commerce	Marine delivery	Single & Multi	NA
[31]	Grocery & Food	Grocery and food	Single	Upto 10 Kg
[32]	Grocery & Food	Pizza	Single	Upto 5 Kg
[33]	Grocery & Food	Taco	Single	NA

## System model

**Fig. 2:**
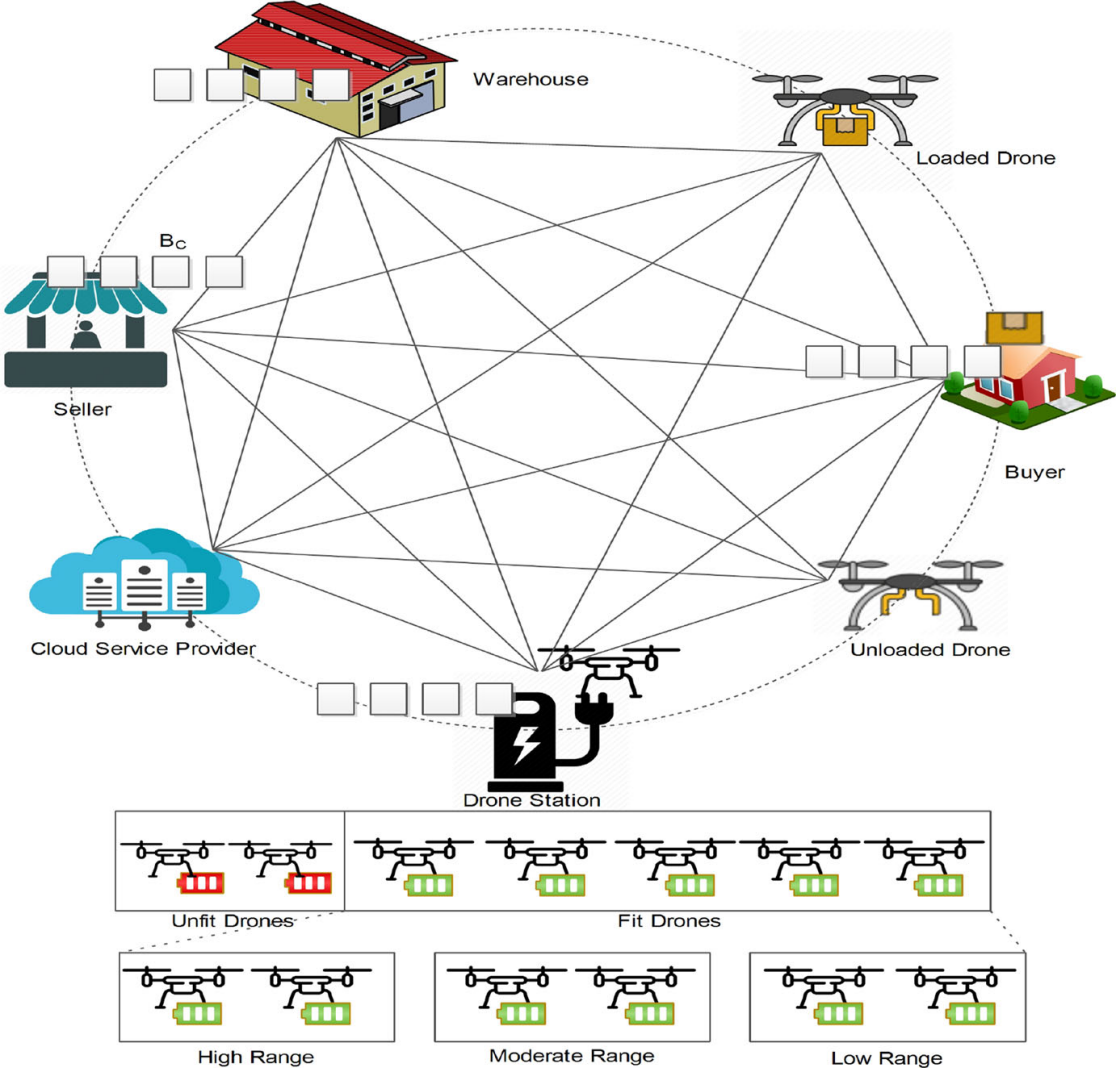
System model for the drone delivery scenario

In this section, the various components of the drone delivery system are discussed. In the system model, blockchain is used to ensure a secure communication environment and smart contracts ensure the trust of various entities (buyer, seller, drone, service provider, etc.). Any change in the system state during the drone delivery process is referenced as a transaction on the blockchain. Figure 2 presents the system model for the drone delivery scenario, various components of which are discussed as follows.

### Seller

In the proposed model, the products are sold by an entity called seller (${\mathbf {S}}$). It provides the details and cost of the products to a cloud-based service provider ($\mathbf {CS}$) for product listing on the online platform. ${\mathbf {S}}$ continuously updates the product information to $\mathbf {CS}$ with respect to its availability in the warehouse (${\mathbf {W}}$).

### Buyer

A buyer (${\mathbf {B}}$) can use the $\mathbf {CS}$ platform to buy any product listed there. ${\mathbf {B}}$ can browse products and finally place the order on the $\mathbf {CS}$ platform. However, before the buyer can actually place the order, a smart contract should be prepared by ${\mathbf {S}}$ which must be accepted by ${\mathbf {B}}$ to avoid any future conflicts. Once the contract is executed, an invoice ($\mathbf {IV}_{{\mathbf {O}}_{k}}$) is generated for the *k*th order (${\mathbf {O}}_k$) placed by *j*th buyer (${\mathbf {B}}_j$) with *i*th seller (${\mathbf {S}}_i$).

### Cloud-based service provider

$\mathbf {CS}$ offers an online merchandise service wherein it lists the products sold by (${\mathbf {S}}$). It is responsible for online product listing and the management of the order received from ${\mathbf {B}}$ in the proposed architecture. It provides middleware services to the ${\mathbf {B}}$ and ${\mathbf {S}}$ in a systematic manner. The key roles of $\mathbf {CS}$ are enlisted below:

- *Product enlisting service* ${\mathbf {S}}$ uses the product enlisting service to list their products on the $\mathbf {CS}$ platform. These products are suggested to ${\mathbf {B}}$ on the online platform as per their interests.
- *Browsing service* ${\mathbf {B}}$ can use the online platform provided by $\mathbf {CS}$ to search, browse and order any product of their choice. The $j^{th}$ buyer (${\mathbf {B}}_j$) can place the order ${\mathbf {O}}_k$ with the $i^{th}$ seller ${\mathbf {S}}_i$.
- *Smart contract* A smart contract ($\mathbf {SC}$) is an auto-initialized code which is executed when certain conditions are met. When ${\mathbf {B}}_j$ wants to buy a product from ${\mathbf {S}}_i$ through the $\mathbf {CS}$, an $\mathbf {SC}$ is created before ${\mathbf {O}}_k$ is placed at the $\mathbf {CS}$. This $\mathbf {SC}$ is signed by both ${\mathbf {B}}_j$ and ${\mathbf {S}}_i$ to generate a corresponding ${\mathbf {O}}_k$.
- *Blockchain* The interactions with the blockchain ($\mathbf {BC}$) are performed by $\mathbf {CS}$. Here, $\mathbf {CS}$ adds the shipping details $\mathbf {SH}_{{\mathbf {O}}_{k}}$ corresponding to ${\mathbf {O}}_k$ on the $\mathbf {BC}$.

### Warehouse

${\mathbf {S}}_i$ stores its products at ${\mathbf {W}}$ and continuously updates the availability status of the product to the $\mathbf {CS}$. When $\mathbf {IV}_{{\mathbf {O}}_{k}}$ is generated by ${\mathbf {S}}_i$ for ${\mathbf {O}}_k$, the process to prepare the product for shipping (delivery) is initiated at ${\mathbf {W}}$.

### Drone station

The drones are responsible for the product delivery from ${\mathbf {W}}$ to the address provided by ${\mathbf {B}}$. The drones are located in the facility called drone station ($\mathbf {DS}$). The flight of $i^{th}$ drone (${\mathbf {D}}_i$) begins from $\mathbf {DS}$ leading towards the shipment pickup from the ${\mathbf {W}}$ and then delivering it to ${\mathbf {B}}_j$ on the address provided in $\mathbf {SH}_{{\mathbf {O}}_{k}}$. After successful shipping of the product(s), ${\mathbf {D}}_i$ moves back to $\mathbf {DS}$. At $\mathbf {DS}$, the drones might be categorised into following categories based on their properties and states.

- *Unfit drones*(${\mathbf {D}}_i^{U}$) When ${\mathbf {D}}_i$ returns from a delivery, the status of the physical resources is monitored by the $\mathbf {DS}$. This status includes battery levels, operations of hardware components, or other physical resources. If the battery status ($\mathbf {BT}_i$) of ${\mathbf {D}}_i$ is below a threshold required for the shortest possible flight in the future, then ${\mathbf {D}}_i$ has to charge its battery to be eligible to join the fit list.
- *Fit drones* (${\mathbf {D}}_i^{F}$) ${\mathbf {D}}_i$ that has all its operational resources in functional order and $\mathbf {BT}_i$ above a threshold value ($\mathbf {BT}_{TH}$), then it is considered to be fit. Algorithm 2 is then used to select a drone for delivery from the fit list.

## Blockchain architecture

In proposed technique similar to [15], blockchain is used by the participating entities for the storage of the information. To ensure the participation of trusted entities only, private blockchain is used. In the private blockchain, only permitted participants can join in the network. Moreover the allowed participants are only allowed to perform the authorized operations. The structural component of $\mathbf {BC}$ where the data is actually stored is known as block [34]. Each block is composed of two parts known as header and trailer. The header has the data management and link information, whereas the trailer stores the data.

### Header part

This part has all the information related to the control and management of the blocks. More details on the components of header are as below:

- *Sequence number* The blocks are created as the time passes by. This means that the block ($\mathbf {BL}_i$) is created after $\mathbf {BL}_{i-1}$ and before $\mathbf {BL}_{i+1}$. The block gets stored in chronological order of their creation with the help of control information field known as sequence number ($\mathbf {SQ}_i$).
- *Timestamp* Blockchain has a problem, known as forking, where the consistent state of blockchain with different entities is different because the main fork is split into different forks. To identify the occurrence of the events, a timestamp ($\mathbf {TS}_i$) of block generation is embedded inside the header.
- *Previous block hash* The biggest advantage that blockchain brings in is the distributed data storage along with the property that transactions once committed are immutable. This is achieved through embedding the hash of the previous block into the new block being made, i.e., in *i*th block, the hash of the $i-1{\mathrm{th}}$ block is stored as previous block hash (${\mathbf {H}}_{\mathbf {BL}_{i-1}}$).
- *Current block hash* Based on the difficulty level, the computed current block hash has certain number of leading bits as zero in the target hash value.
- *Nonce* The hash of the current block can only be achieved if we set a value inside the header that forces the output hash to be of specific nature. It is achieved using pseudo random numbers called nonce (${\mathbf {N}}$).

### Trailer part

The area where the data is stored inside the block is known as trailer part. The trailer in the proposed blockchain model comprises the following:

- *Smart contract* When ${\mathbf {B}}$ places an order, it is converted into $\mathbf {SC}$ which is signed by ${\mathbf {S}}$ and ${\mathbf {B}}$ as an agreement.
- *Shipping* If the shipping information is manipulated by an attacker, the product may be delivered to the wrong address. Hence, to keep the shipping information immutable, it is stored on the blockchain.

The structure of $\mathbf {BC}$ is appropriate for all entities except the drone due to its resource limitations. If the complete blockchain is stored on a drone, it may lead to the over whelming of drone resources. Thus, a decoupled blockchain ($\mathbf {BC}^{\mathbf {D}}$) is used for drones and $\mathbf {GS}$ communications. The data and header parts are decoupled from the blockchain to keep the storage requirement on drones to the minimum. The $\mathbf {BC}^{\mathbf {D}}$ comprises only the header part of different blocks. Moreover, each drone has its own block which is appendable in nature. This way every time new data is added to the block of the drone, the resulting hash of the data block changes. The hash of the data of drones is embedded into the blockchain of headers, i.e., $\mathbf {BC}^{\mathbf {D}}$. The blockchains are maintained with the $\mathbf {CS}$, while the drones store only their own block excluding the data of other drones within the $\mathbf {BC}^{\mathbf {D}}$. The block structure used in $\mathbf {BC}^{\mathbf {D}}$ for drone-to-drone and drone-to-ground station communication, and data storage differs from the $\mathbf {BC}$ in the following aspects:

- *Header* In the decoupled blockchain, the header contains the control information for ${\mathbf {D}}_i$. This includes the hash of previous block, drone attributes such as payload capacity (${\mathbf {D}}_{pld}^{max}$), ratted battery capacity (${\mathbf {D}}_{bat}^{rtd}$), hash of drones registration identity (${\mathbf {H}}_{{\mathbf {D}}_{id}}$), timestamp of block header creation (${\mathbf {T}}^{hdr}$), drones public key (${\mathbf {D}}_i^{pb}$) and private key (${\mathbf {D}}_i^{pr}$), and the merkle root hash ($\mathbf {MRH}$) of the drone data (${\mathbf {D}}_{i}^{MRH}$).
- *Data* The data is not stored on the blockchain and only the $\mathbf {MRH}$ of the data is stored inside the header to ensure integrity. However, the $\mathbf {DS}$ and $\mathbf {CS}$ maintain the complete copy of data and header for drone interactions, while the drones store the current mission-related data (${\mathbf {D}}_{i(d)}^{cur})$ only.

## CovaDel: the proposed drone delivery framework

The proposed framework for drone-based delivery scenario consists of several phases that work in tandem to achieve the overall objective. These phases are discussed in the following sections.

### Initial agreement and smart contract creation phase

**Fig. 3:**
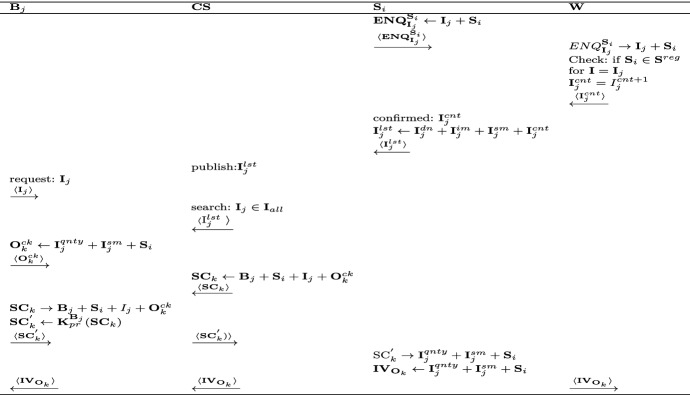
Initial agreement and smart contract creation

The first phase deals with establishing an agreement between the ${\mathbf {B}}$ and ${\mathbf {S}}$. This agreement is translated in to a smart contract that is deployed on the blockchain. The actions performed in this phase are depicted in Fig. 3.

- ${\mathbf {S}}_i$ sends an enquiry ($\mathbf {ENQ}_{{\mathbf {I}}_{j}}^{{\mathbf {S}}_{i}}$) to ${\mathbf {W}}$ for the available quantity of the item (${\mathbf {I}}_j$).
- When ${\mathbf {W}}$ receives $\mathbf {ENQ}_{{\mathbf {I}}_{j}}^{{\mathbf {S}}_{i}}$, it authenticates ${\mathbf {S}}_i$ from the list of registered sellers (${\mathbf {S}}^{reg}$) and the following condition should be true.
  1 $$\begin{aligned} {\mathbf {S}}_i \in {\mathbf {S}}^{reg} \end{aligned}$$
  After this process, ${\mathbf {W}}$ check the ${\mathbf {I}}_j$ in the stock and reverts ${\mathbf {S}}_i$ with available stock inventory count (${\mathbf {I}}_j^{cnt}$) for the enquired item.
- Based on ${\mathbf {I}}_j^{cnt}$, the ${\mathbf {S}}_i$ lists the items on $\mathbf {CS}$ for sales. The $j{\mathrm{th}}$ listed item (${\mathbf {I}}_j^{lst}$) details on $\mathbf {CS}$ comprises of item description (${\mathbf {I}}_j^{dn}$), images (${\mathbf {I}}_j^{im}$), available shipping methods (${\mathbf {I}}_j^{sm}$) and the ${\mathbf {W}}$ confirmed stock count (${\mathbf {I}}_j^{cnt}$). The item listing (${\mathbf {I}}^{lst}$) on the $\mathbf {CS}$ includes the following details.
  2 $$\begin{aligned} {\mathbf {I}}_j^{lst} \rightarrow \{{\mathbf {I}}_j^{dn} + {\mathbf {I}}_j^{im} + {\mathbf {I}}_j^{sm} + {\mathbf {I}}_j^{cnt}\} \end{aligned}$$
- ${\mathbf {B}}_j$ search for required ${\mathbf {I}}_j$ at $\mathbf {CS}$ platform and place the request. On the receipt of request, $\mathbf {CS}$ checks ${\mathbf {I}}_j$ in the available items and revert ${\mathbf {B}}_j$ with confirmation.
- Now, ${\mathbf {B}}_j$ proceeds with the order (${\mathbf {O}}_k^{ck}$) wherein it mentions the quantity (${\mathbf {I}}_j^{qnty}$), shipping method (${\mathbf {I}}_j^{sm}$) and preferred ${\mathbf {S}}_i$. The generated ${\mathbf {O}}_k^{ck}$ is confirmed for further processing with $\mathbf {CS}$.
- Once $\mathbf {CS}$ receives ${\mathbf {O}}_k^{ck}$, it prepares $\mathbf {SC}_k$ (for $k{\mathrm{th}}$ order) comprising information about ${\mathbf {B}}_j$, ${\mathbf {S}}_i$, ${\mathbf {I}}_j$ and ${\mathbf {O}}_k^{ck}$ and then send it to ${\mathbf {B}}_j$ for final approval.
- Now, ${\mathbf {B}}_j$ signs the $\mathbf {SC}_k$ using its private key (${\mathbf {K}}_{pr}^{{\mathbf {B}}_j}$) and send to $\mathbf {CS}$ as follows:
  3 $$\begin{aligned} \mathbf {SC}_k^{'} \leftarrow {\mathbf {K}}_{pr}^{{\mathbf {B}}_j} (\mathbf {SC}_k) \end{aligned}$$
- $\mathbf {CS}$ send the $\mathbf {SC}_k^{'}$ to ${\mathbf {S}}_i$ for preparation of invoice($\mathbf {IV}_{{\mathbf {O}}_{k}}$). Finally, the $\mathbf {IV}_{{\mathbf {O}}_{k}}$ is sent to the ${\mathbf {B}}_j$ through $\mathbf {CS}$.

### Delivery phase

**Fig. 4:**
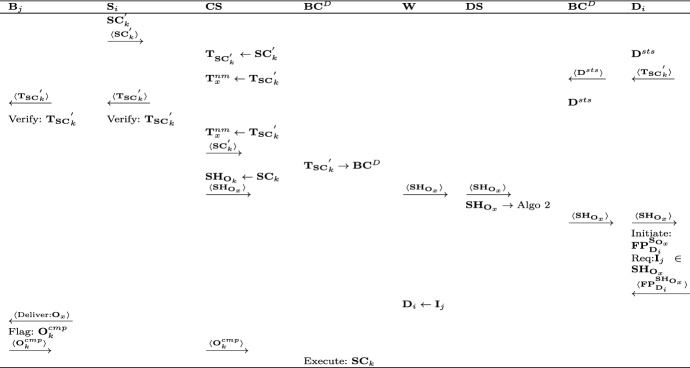
Delivery phase

The second phase deals with the delivery of the product from ${\mathbf {W}}$ to the buyers address. The actions of the involved entities are as shown in Fig. 4.

- $\mathbf {CS}$ adds $\mathbf {SC}_{k}$ into the $\mathbf {BC}$. Initially, $\mathbf {SC}_{k}$ is added to the pool of un-mined transactions (${\mathbf {T}}_x^{nm}$) in the form of ${\mathbf {T}}_{\mathbf {SC}_{k}}$.
  4 $$\begin{aligned} {\mathbf {T}}_x^{nm} \leftarrow {\mathbf {T}}_{\mathbf {SC}_{k-1}} + {\mathbf {T}}_{\mathbf {SC}_{k}} + {\mathbf {T}}_{\mathbf {SC}_{k+1}} +\cdots {\mathbf {T}}_{\mathbf {SC}_{k+n}} \end{aligned}$$
- ${\mathbf {B}}$ and ${\mathbf {S}}$ act as miners on the $\mathbf {BC}$. The transaction between ${\mathbf {B}}_j$ and ${\mathbf {S}}_i$, i.e., ${\mathbf {T}}_{\mathbf {SC}_{k}}$ is verified and further sent for mining into the block of $\mathbf{BC}$. If either of the involved entities can’t verify ${\mathbf {T}}_{\mathbf {SC}_{k}}$, it is denied and aborted.
- Once the ${\mathbf {T}}_{\mathbf {SC}_{k}}$ becomes part of $\mathbf {BC}$, the $\mathbf {SH}_{{\mathbf {O}}_{k}}$ is extracted from it and provided to $\mathbf {DS}$. Now, $\mathbf {DS}$ selects and schedules ${\mathbf {D}}_i$ using Algorithm 2.
- $\mathbf {DS}$ receives an up to date information about drone status through $\mathbf {BC}^D$. The $\mathbf {SH}_{{\mathbf {O}}_{k}}$ is added to the block of ${\mathbf {D}}_i$ in $\mathbf {BC}^D$.
- ${\mathbf {D}}_i$ reads the information from its block on $\mathbf {BC}^D$ and initiates a flight plan ($\mathbf {FP}$) for the execution of ${\mathbf {O}}_k$ ($\mathbf {FP}_{{\mathbf {D}}_{i}}^{\mathbf {SH}_{{\mathbf {O}}_{k}}}$).
- ${\mathbf {D}}_i$ collects the ${\mathbf {I}}_j$ as per ${\mathbf {O}}_k$ from ${\mathbf {W}}$ and delivers it as per $\mathbf {SH}_{O_{k}}$ and return to the $\mathbf {DS}$ where $\mathbf {DS}$. Now, the state of ${\mathbf {D}}_i$ is updated on the $\mathbf {BC}^D$ after delivery.
- ${\mathbf {D}}_i$ adds its status (${\mathbf {D}}^{sts}$) comprising of drone payload capacity (${\mathbf {D}}_{pld}^{max}$), rated battery capacity(${\mathbf {D}}_{bat}^{rtd}$), hashed identity(${\mathbf {D}}_{id}^{hash}$) and the public private key pair ($\mathbf{K }_{pr}^{{\mathbf {D}}_i},\mathbf{K }_{pb}^{{\mathbf {D}}_i}$) on $\mathbf {BC}^D$. This information acts as input to the Algorithm 2 using multi-level queuing technique to schedule ${\mathbf {D}}_i$ for a delivery.
- When ${\mathbf {B}}_j$ receive ${\mathbf {I}}_j$ as per ${\mathbf {O}}_k$, a flag (${\mathbf {O}}_k^{cmp}$) is raised confirming the receipt of ${\mathbf {I}}_j$. The $\mathbf {SC}_{k}^{'}$ is executed by $\mathbf {BC}^D$ and the status is notified to ${\mathbf {S}}_i$.

### Payment phase

**Fig. 5:**
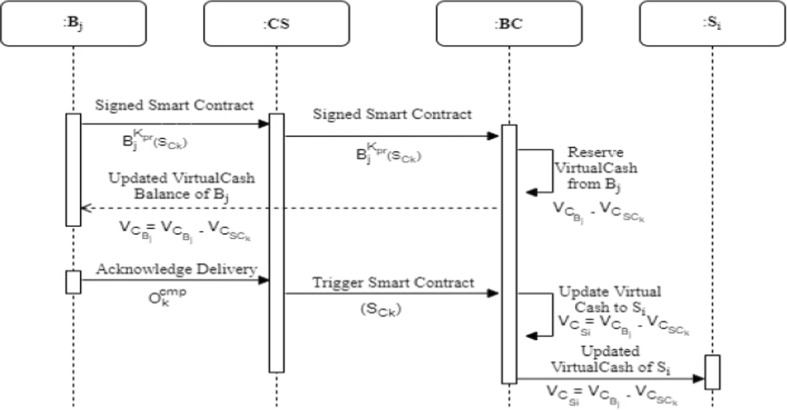
Payment phase

The third phase is related to the payment to the ${\mathbf {S}}_i$ from the ${\mathbf {B}}_j$’s reserve. The $\mathbf {SC}$ is auto executed once the order is delivered to ${\mathbf {B}}_j$ and the corresponding amount is transferred to the ${\mathbf {S}}_i$’s cash wallet. The interaction between various entities is shown in Fig. 5.

- ${\mathbf {B}}_j$ agrees to the $\mathbf {SC}$ made by the $\mathbf {CS}$ by signing it, i.e., $\mathbf {SC}_k^{'}$ and the same is forwarded to the $\mathbf{BC}$ through $\mathbf {CS}$.
- On receiving $\mathbf {SC}_k^{'}$, the $\mathbf{BC}$ reserves the amount of virtual cash ($\mathbf{VC} _{{\mathbf {B}}_{j}}$) corresponding to the $\mathbf {SC}_{k}$ from the available virtual cash ($\mathbf {VC}_{{\mathbf {B}}_{j}}$) of ${\mathbf {B}}_j$.
- The updated $\mathbf {VC}_{{\mathbf {B}}_{j}}$ is returned to ${\mathbf {B}}_j$ to eliminate the double spending problem, as ${\mathbf {B}}_j$ is not able to spend the reserved amount until ${\mathbf {O}}_k$ either succeeds.
- Once ${\mathbf {I}}_j$ is delivered by ${\mathbf {D}}_i$ to ${\mathbf {B}}_j$, it initiates the order completion flag (${\mathbf {O}}_k^{cmp}$).
- On receiving ${\mathbf {O}}_k^{cmp}$, the $\mathbf {SC}_{k}$ is triggered on the $\mathbf {BC}$ that adds $\mathbf {VC}_{\mathbf {SC}_{k}}$ to the Virtual cash($\mathbf {VC}_{{\mathbf {S}}_{i}}$) of ${\mathbf {S}}_i$.
- The updated $\mathbf {VC}_{{\mathbf {S}}_{i}}$ is returned to the ${\mathbf {S}}_i$ leading to the completion of payment.
  5 $$\begin{aligned} \mathbf {VC}_{{\mathbf {S}}_{i}} = \mathbf {VC}_{{\mathbf {S}}_{i}} + \mathbf {VC}_{\mathbf {SC}_{k}} \end{aligned}$$

### Multi-queue drone indexing phase

Initially, the drones are categorized into fit and Un-fit drone queue based on their current battery status. Further, the available drones are segregated into the heavy range queue (${\mathbf {Q}}^{HR}$), moderate range queue (${\mathbf {Q}}^{MR}$), and low range queue (${\mathbf {Q}}^{LR}$) using the proposed multi-queue drone indexing algorithm (Algorithm 1).
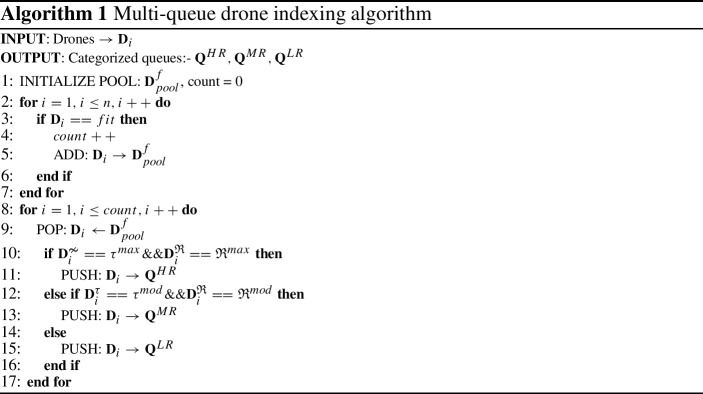


Various parameters are considered for categorization of the drones like, towing capacity (${\mathbf {D}}_i^{{\tau }}$), battery status (${\mathbf {D}}_i^{\mathfrak {B}}$), and maximum coverage range (${\mathbf {D}}_i^{\mathfrak {R}}$). The working of this approach is discussed in the below mentioned steps:

- Initially, all the fully charged (fit) drones are pushed into the pool (${\mathbf {D}}^f_{pool}$) starting with an initial count = 0.
- Further, the drones are selected for segregation from the ${\mathbf {D}}^f_{pool}$.
- If ${\mathbf {D}}_i^{{\tau }}$ towing capacity of the drone is maximum ($\tau ^{max}$), ${\mathbf {D}}_i^{\mathfrak {R}}$ range is maximum ($\mathfrak {R}^{max}$), then push the ${\mathbf {D}}_i$ to the ${\mathbf {Q}}^{HR}$ queue.
- If ${\mathbf {D}}_i^{{\tau }}$ towing capacity of the drone is moderate ($\tau ^{mod}$), ${\mathbf {D}}_i^{\mathfrak {R}}$ range is narrow ($\mathfrak {R}^{mod}$), then index the ${\mathbf {D}}_i$ to the ${\mathbf {Q}}^{MR}$ queue.
- Otherwise, push the ${\mathbf {D}}_i$ to the ${\mathbf {Q}}^{LR}$ queue.

In the algorithm, $\tau ^{max}$, and $\tau ^{mod}$ are variable and can be fixed based on the drone technology. Similarly, $\mathfrak {R}^{max}$, and $\mathfrak {R}^{mod}$ are also variable and can be fixed based on a specific drone model. The proposed approach helps to select a suitable drone for the delivery. The queue manager supervise all the queues and update the status of all the loaded and unloaded drones in the defined queues. Further, the algorithmic complexity is tight upper bound defined as $\theta (n)$.

### Drone scheduling phase

$\mathbf {GS}$ is assigned with the task of optimal selection of drones for shipping the product (${\mathbf {O}}_k$) to the delivery address. The $\mathbf {CS}$ provides the list of the requested products ${\mathbf {S}}_{O_{k}}$ to the $\mathbf {GS}$ for further processing. The number of orders (${\mathbf {O}}_k$) initiated in a short interval of time are combined into one optimised flight by the proposed scheduling algorithm. The detailed working of the model is discussed below:

- Scheduling algorithm accepts the input as ${\mathbf {S}}_{O_{k}}$ from the $\mathbf {CS}$ comprising of ${\mathbf {O}}_k$’s weight (${\mathbf {O}}^{wt}_k$), dimensions of package (${\mathbf {O}}^{di}_k$), destination location coordinates (${\mathbf {O}}^{dest}_k$), nature of shipment (${\mathbf {O}}^{type}_k$) and delivery timing (${\mathbf {O}}^{dt}_k$) and **TP** are the total number of products to ship at various locations.
  6 $$\begin{aligned} {\mathbf {S}}_{O^\mathbf{TP }_k} \leftarrow \mathbf {CS}_{(\mathbf {O_{k}^{wt}}, \mathbf {O_{k}^{di}}, \mathbf {O_{k}^{dest}}, \mathbf {O_{k}^{type}},\mathbf {O_{k}^{dt}} )} \end{aligned}$$
- Fetch ${\mathbf {Q}}^{HR}, {\mathbf {Q}}^{MR}, {\mathbf {Q}}^{LR}$ from Algorithm 1.
- The newly initiated ${\mathbf {S}}_{O_{k}}$ pushed into a shipping queue ($\mathbf {SQ}$) as ${\mathbf {S}}_{O_k} \rightarrow \mathbf {SQ}$
- After the shipping requests are pushed into $\mathbf {SQ}$, the following steps are performed till all the shipping request in $\mathbf {SQ}$ are served.
  - The $\mathbf {SQ}$ is mapped with the ${\mathbf {Q}}^{HR}, {\mathbf {Q}}^{MR}, {\mathbf {Q}}^{LR}$ for selection of a drone.
  - The number of drones (${\mathbf {N}}$) from the matched queue are further mapped as per ${\mathbf {O}}_k$ requirements.
  - The best match (${\mathbf {D}}_i$) is the one with minimum difference in ${\mathbf {D}}^{cap}_i$ and ${\mathbf {S}}_{O_{k}}$.
  - The selected ${\mathbf {D}}_i$ is allocated to ${\mathbf {O}}_k$ for product delivery.
  - The details of the ${\mathbf {S}}_{O_{k}}$ is offloaded to ${\mathbf {D}}_i$ for flight.
  - After successfully delivery of ${\mathbf {O}}_k$, it is removed from the $\mathbf {SQ}$ of the $\mathbf {DS}$.
- The approach return the mapped ${\mathbf {D}}_i \rightarrow {\mathbf {O}}_k$ to the ${\mathbf {W}}$, which further hands over the ${\mathbf {O}}_k$ to the ${\mathbf {D}}_i$ for delivery to the assigned location.

The distance of the destination ${\mathbf {B}}_j$ from the warehouse ${\mathbf {W}}$ is computed using the following equation as suggested in [34]:

7 $$\begin{aligned} {d}_{({\mathbf {w}} \rightarrow {\mathbf {B}}_j)} = \left| \frac{{d}_{\mathbf {W}}}{{d}}\right| \times {d} + \left| \frac{d_{{\mathbf {B}}_{j}}}{d}\right| \times d + n_{({\mathbf {W}}\rightarrow {\mathbf {B}})} \times d \end{aligned}$$

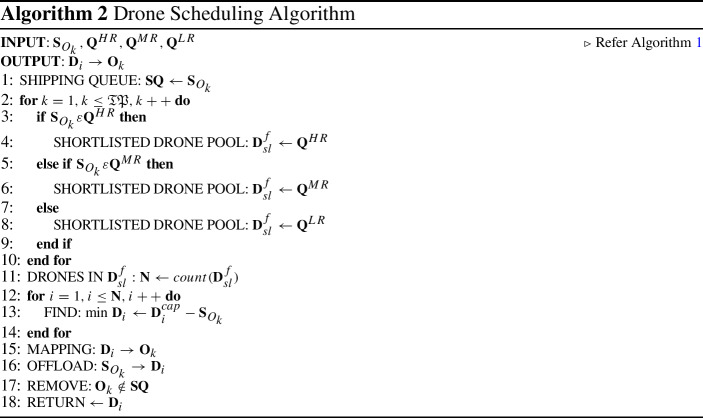


The algorithmic complexity of this algorithm is calculated as $O(TP+N)$.

## QoS-aware communication approach

CovaDel is strictly dependent on timely data/information from the participating entities. At a given time, multiple drones may be out for delivery (or other missions) so they can communicate among themselves and to the $\mathbf {GS}$ to pass the vital information (like, drone density or critical incident in the region) that can aid the drone-based delivery process. The density of the drones flying in a given region can lead towards the bandwidth fluctuations thereby limiting availability of the communication resources. This can further end up in network collisions and thereby network congestion. Now, the collisions and congestion in the network can eventually effect the QoS metrics and therefore degrade the QoE for the drone delivery scenario. The proposed QoS-aware communication approach helps to establish a connection between any two drones for sharing prompt information while adhering to the QoS metrics (end-to-end delay and network throughput). The aims of this approach are concerned with the collision avoidance and congestion control in order to to provide adequate QoS for the underlying communications related to drone delivery process. This approach adopts accurate positioning beacons for collision avoidance among drones and then apply congestion-avoidance policy to coordinate the communication.

This approach uses the properties of fire-fly optimization algorithm [35] and proposes an efficient mechanism based on light-intensity formation (an attractiveness parameter) to enhance QoS in drone communications. Firefly algorithm considers the concept of flashing light (produced through the process of bio-luminescence) generated by fire flies and correlate it to signaling systems. The fundamental functions related to the fire fly’s flashing light, also known as attractiveness functions include, (a) to attract mating partners, and (b) to attract prey. Additionally, the flash light is also used as a signal for protective warning. The rate, pattern and related time are key aspects of flashing light that form a part of signalling system between fire flies. The flashing light concept has been idealized and associated with objective functions related to optimization to formulate a firefly algorithm in [35]. This algorithm follows three key rules, (a) all fireflies are unisex and they can attract each other irrespective of their sex (ideally fit in case of drones), (b) attractiveness is correlated to brightness and both decrease with an increase in the distance, and (c) the brightness can vary with respect to the representation of the objective function. Further, this algorithm relies on two key concepts, (a) variation in the light intensity, and (b) formulation of attractiveness. Looking in to these two factors, the attractiveness of a firefly can be decided on the basis of its brightness (higher or lighter light intensity) and thereby linked to an objective function. We have applied this concept for drone communications and defined two objectives related to collision avoidance and congestion control based on the light intensity (brightness) that varies with an increase in the distance between the drones in a given area.

Inspired form the above concept, a communication approach has been designed wherein we have used the time slot mechanism proposed in [14] to form a connection between drones. Based on this, the proposed approach establish collision avoidance strategy through accurate position beacons as suggested in [36] and thereafter use the congestion control policy to realize the communication. The positioning of the beacons is vital and the effect of localization of drones is critical for drone delivery scenario. As drones have high mobility, the anchor points for the localization tend to change and finding appropriate anchor points is crucial. The proposed technique uses the light intensity parameter for optimization of the drone communications, that relies on the effective beacon positioning and hence provides effectiveness in areas with no fixed infrastructure to deploy the congestion avoidance policies. On the other side of the coin, the beacon position impacts the efficiency of the congestion avoidance which is susceptible to fail if the beacons are not chosen effectively.

In this approach, the average-light intensity received by the drones positioned at location $L_i$ and $L_j$ becomes the base of the proposed collision avoidance approach. In this regard, we define the average-light intensity (${\mathcal {l}}^{(r)}_{A, i}$) received by the drones as below [14].

8 $$\begin{aligned} {\mathcal {l}}^{(r)}_{(i, j)}=\alpha _{i}^{(t)}+\alpha _{(i,0)}^{(t)} e^{-\eta \beta ^{2}} \left( L_{i}-L_{j}\right) +\gamma \end{aligned}$$

where, the attraction value between two drones concerning *i* *rmth* is denoted by $\alpha _{i}^{(t)}$, initial attraction value is represented by $\alpha _{i,0}^{(t)}$, $\gamma$ denotes the density of a drones in its neighbourhood, $\eta$ represents the rate of change with respect to the present route, and inverse of probability of connectivity is represented by $\beta$.

${\mathcal {l}}^{(R)}_{A, i}$ is computed for all the drones in the region, where $\gamma$ act as the varying factor to enforce the varied number of inter-connected drones. Based on location-awareness, this model identifies collision possibilities as follows.

9 $$\begin{aligned} {\mathcal {l}}^{(r)}_{i,j} \le \left( {\mathcal {l}}^{(r)}_{i,j}\right) _{(THR)} = \min \left( \overline{{\mathcal {l}}^{(r)}_{i}}\right) \forall D \end{aligned}$$

where, *THR* represents a threshold and *D* represents a set of drones.

The work pattern of the original firefly algorithm tends to increase the light intensity (attractiveness or brightness) that doesn’t fit with the requirement of the proposed model. The requirement of this approach is concerned with the lower light intensity as higher intensity (brightness) depicts the possibility of a collision. Thus, we used the modified firefly algorithm from [14] that operates in a reverse pattern thereby triggering the collision avoidance mechanism within the defined time cycle in an efficient manner.

Once the collision possibilities are identified, the next task relates to the congestion control in the network. For this purpose, for the incoming traffic, the intensity mechanism (of firefly algorithm) is used as the base to decrease (or increase) the congestion window by using lower (or higher) streaks for any given connection as proposed in [14]. Let us say, the traffic rate between any two drones is (${\mathcal {V}}_{i,j}$), then the congestion-control mechanism works for both drones in a simultaneous manner while considering (or managing) their transmissions ($\tau$) and receptions. The congestion-control evaluations can be expressed as below.

10 $$\begin{aligned} {\mathcal {C}}^{(r)}_{i,j}={\mathcal {V}}_{i,j}^{(t)}+{\mathcal {V}}_{o} e^{-v \theta ^{2}} \varDelta C + C_(cp) \end{aligned}$$

where, v denotes the velocity of the drone, $\theta$ represents the current heading of a drone, $\varDelta C$ is the possibility of a connection between the drones and ranges between 0 and 1 for any incoming drones, and $C_(cp)$ represents the number of channels for the connected components in average connectivity in a network.

In the above defined congestion-control evaluation, if at an instance there is an increase in the value of ${\mathcal {C}}^{(r)}_{i}$, then the network may end up in congestion. Thus, the proposed model need to adjust traffic based on the following condition.

11 $$\begin{aligned} {\mathcal {C}}^{(r)}_{i} \le \big ({\mathcal {C}}^{(r)}_{i}\big )_{THR} \end{aligned}$$

where, *THR* represents the threshold that is based on the average rate that is sustainable over a defined number of channels.
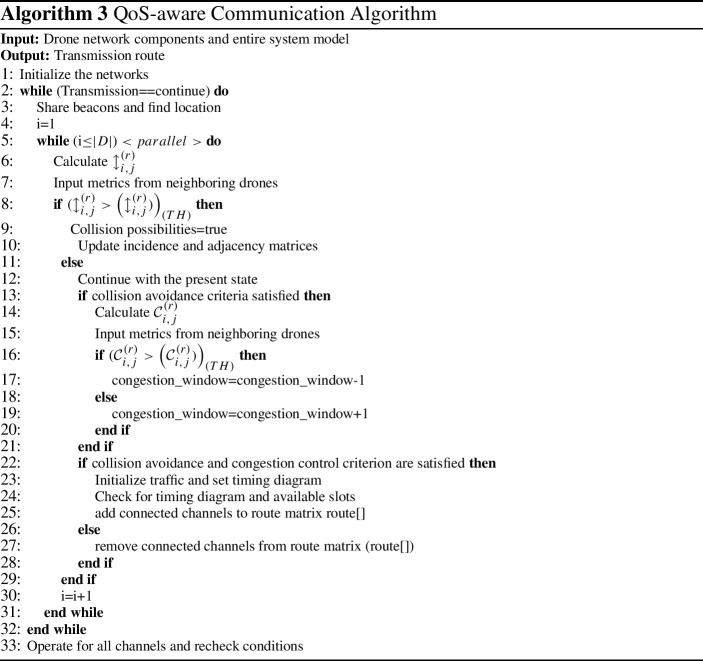


Based on the above evaluations, the Algorithm 3 represents the workflow of the QoS-aware drone communications. Here, the first step involves satisfying the collision avoidance criteria. The mechanism continuously checks the possibilities of collisions over the defined way-points thereby averting any possible termination in the transmission. The mechanism ensures that a drone must satisfy the defined light-intensity constraints keeping in view of the neighbouring drone. If in a case, these conditions are not satisfied or doesn’t hold TRUE, then an instant feedback is triggered so that the timing control can consider it while providing a time slot for next drone communication.

Once the collision-avoidance possibilities are satisfied, the next step includes the fulfilment of congestion control evaluations. The algorithm checks the possible collisions over the way-points in a continuous manner by managing the size of the congestion window. This helps to prevent any unintentional breakage in the transmissions and traffic overloading scenario. Once both the criterion are satisfied, we proceed to check for route while maintaining conditions related to the QoS requirements. The overall algorithmic complexity of this algorithm is $O(D*\tau )$.

## Experimental evaluation and discussion

The proposed scheme is evaluated on the basis of three directions, (1) blockchain validation for smart contracts, (2) numerical validation, and (3) QoS validation based on simulation experiments.

### Blockchain validation using ethereum-based test network

The smart contacts are used in the proposed framework to ensure the integrity of the system resulting in trust of the involved entities. The experimental setup for validation is elaborated below.

- *Environment setup* The logic used in the proposed scheme is translated in the form of smart contacts, which are executed through remix IDE [37] in combination with web3 scripting environment. The performance is evaluated on an Ethereum-based test network [38]. Metamask [39] account has been setup to access the Ropsten test network wherein the smart contacts are deployed. This environment is setup on a personal machine with a fixed hardware configuration configuration (Intel i7 7700HQ, 16GB RAM, PC4-19200 DDR4 2400MHz, Overclocked to 3.8GHz, Nvidia GeForce GTX 1060 (4GB)).

**Fig. 6:**
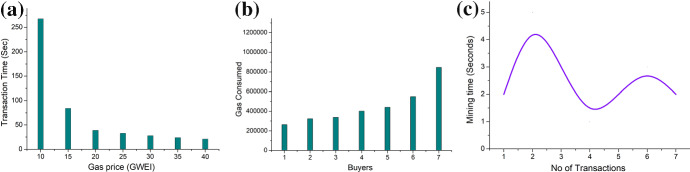
**a** Gas price versus transaction time, **b** Gas consumed versus number of buyers, and **c** No of transactions versus mining time

The effectiveness of the smart contracts, which is regarded as mileage according to Ethereum terminology, is evaluated through the price paid for the gas for deployment of the smart contracts. The first tested smart contact is for the interaction between the buyer and the seller. The results obtained are depicted in Fig. 6a, suggesting the effect of the paid gas price on the transaction throughput. With more cost paid for gas per unit transaction, the transaction throughput increases. The improvement ratio in transaction though is more around the lower end, whereas moving towards expensive transactions does not improve the throughput proportionately. Hence, to meet the requirements of the QoS, the gas amount has to be chosen accordingly to control the deployability of the proposed approach. Next, we validated the effect of the consecutive number of buyers in the proposed framework on the amount of gas consumption for smart contract execution. The findings are depicted in Fig. 6b, representing the trend that with an increase in the number of transactions, the gas requirement and hence the cost of deployment tends to increase in a controlled manner. In the end, the model is validated for the mining time with respect to the number of transactions that are simultaneously committed and the results are depicted in Fig. 6c. The results suggests the non linear variation of mining time which can be attributed to the miners. The possible explanations for the variation include the nonce whose computation is probabilistic in nature. Sometime nonce is computed very quickly, whereas sometimes it takes a longer set of combinations to be tried by the miners before a successful resultant. These validations give valuable insights into the resource consumption and the load of computation related to CovaDel.

### Numerical validation based on computation and communication costs

The proposed model witness number of interactions between different parties. So, we compute the computational and communication costs related to these parties.

#### Computational cost

The computational cost associated for each phase is calculated as below.

- *Initial agreement and smart contract phase* In the initial agreement phase, four entities ${\mathbf {B}}_j$, ${\mathbf {S}}_i$, $\mathbf {CS}$ and ${\mathbf {W}}$ are involved. ${\mathbf {B}}_j$ perform 5 append operations taking 0.6 ms in total and one digital signature computation which includes the encryption of $\mathbf {SC}_{k}$ using the private key of buyer costing 1.2 ms. $\mathbf {CS}$ performs one search operation requiring at least 2 ms which tends to increase with the larger database and 4 append operations requiring 0.45 ms. ${\mathbf {S}}_i$ requires 3 append operations to generate the invoice requiring 0.3ms. Lastly, the ${\mathbf {W}}$ performs one search operation requiring 2 ms. Hence, the total computational cost in this phase is 6.55 ms.
- *Delivery phase* In this phase, ${\mathbf {B}}_j$ performs one comparison operation to check the transaction from ${\mathbf {T}}_{\mathbf {SC'}_{k}}$ requiring 0.5ms. When the order gets delivered, ${\mathbf {B}}_j$ needs to generate ${\mathbf {O}}_k^{cmp}$ to indicate the receipt of product which requires 0.2 ms. ${\mathbf {S}}_i$ needs to do one comparison operation in order to validate the ${\mathbf {T}}_{\mathbf {SC'}_{k}}$ which is to be added to the blockchain requiring 0.5 ms. $\mathbf {CS}$ adds the ${\mathbf {T}}_{\mathbf {SC'}_{k}}$ of $\mathbf {SC}_{k}$ into blockchain for which it first adds it into the ${\mathbf {T}}^{nm}_x$ requiring 0.2 ms. After verification by ${\mathbf {B}}_j$ and ${\mathbf {S}}_i$, the ${\mathbf {T}}_{\mathbf {SC'}_{k}}$ is finally mined into the block requiring 2 ms and added to the $\mathbf {BC}^D$. Then, the extraction of $\mathbf {SH}_{{\mathbf {O}}_{x}}$ from the smart contract requires 0.3 ms. The smart contract is added to the blockchain requiring 1 ms. After delivery the smart contract is executed by the blockchain requiring 2 ms. $\mathbf {DS}$ executes the drone scheduling algorithm for which 5 comparison operations are performed requiring 2.5 ms, 6 addition operations requiring 1.8 ms, and searching requiring 0.5 ms. ${\mathbf {D}}_i$ collects the information from $\mathbf {BC}^D$ and then processes the flight plan requiring 2 ms. It also updates its status on the $\mathbf {BC}^D$ which needs 0.4 ms. Cumulatively, the computational cost of the delivery phase accounts to 12.9 ms.
- *Payment phase* The final phase of payment processing requires the $\mathbf {BC}$ to perform one subtraction operation requiring 0.3 ms and one addition requiring 0.3 ms. The $\mathbf {CS}$ needs to perform the trigger on $\mathbf {SC}_{k}$ requiring 0.2 ms. Hence, the total cumulative computational cost of the payment phase is 0.8ms.

This brings the total computational cost of all the three phases to 20.25 ms.

#### Communication cost

The communication cost for different phases is calculated as below:

- *Initial agreement and smart contract phase* The first phase requires the communication between ${\mathbf {B}}_j$ and $\mathbf {CS}$ for ${\mathbf {O}}_k^{ck}$ which is of 39 bits with 32 bits for ${\mathbf {S}}_i$, 4 bits for ${\mathbf {I}}_j^{qnty}$ and 3 bits for ${\mathbf {I}}_j^{sm}$. When the $\mathbf {CS}$ prepares the $\mathbf {SC}_{k}$, it comprising 32 bits ${\mathbf {B}}_j$, 32 bits ${\mathbf {S}}_i$, 32 bits ${\mathbf {I}}_j$ and 39 bits ${\mathbf {O}}_k^{ck}$. Hence, 135 bits are transmitted by the $\mathbf {CS}$. ${\mathbf {S}}_i$ prepares the $\mathbf {IV}_{{\mathbf {O}}_{x}}$ comprising of ${\mathbf {I}}_j^{qnty}$ of 4 bits, ${\mathbf {I}}_j^{sm}$ of 3 bits and 32 bits ${\mathbf {S}}_i$. Hence, these 39 bits are transmitted by ${\mathbf {S}}_i$. Hence, the total communication cost involved in this phase is 213 bits.
- *Delivery phase* Here, $\mathbf {CS}$ sends 135 bit ${\mathbf {T}}_{\mathbf {SC'}_{k}}$ to the ${\mathbf {B}}_j$ and ${\mathbf {S}}_i$ for verification of transaction. ${\mathbf {S}}_i$ and ${\mathbf {B}}_j$ flags the validity of ${\mathbf {T}}_{\mathbf {SC'}_{k}}$ via 1 bit response each. Afterwards, the 135 bit ${\mathbf {T}}_{\mathbf {SC'}_{k}}$ is sent to $\mathbf {BC}^D$. Next, $\mathbf {CS}$ extracts $\mathbf {SH}_{{\mathbf {O}}_{x}}$ from $\mathbf {SC}_{k}$ which is of 64 bits. This 64 bits $\mathbf {SH}_{{\mathbf {O}}_{x}}$ is sent to ${\mathbf {W}}$ and $\mathbf {DS}$. $\mathbf {DS}$ sends the 64 bits $\mathbf {SH}_{{\mathbf {O}}_{x}}$ to ${\mathbf {D}}_i$ chosen drone drone scheduling algorithm. Finally, the ${\mathbf {B}}_j$ sends the ${\mathbf {O}}_k^{cmp}$ to $\mathbf {CS}$ which is of 9 bit including 8 bits for hash of $\mathbf {SC}_{k}$ and 1 bit for indicating the completion. These 9 bits ${\mathbf {O}}_k^{cmp}$ is further sent by $\mathbf {CS}$ to $\mathbf {BC}$ which accounts to 354 bits.
- *Payment phase* This phase comprises of $\mathbf {BC}$ reserving $\mathbf {VC}$ corresponding to $\mathbf {SC}_{k}$ from ${\mathbf {B}}_j$’s $\mathbf {VC}$ and sends 32-bit balance ($\mathbf {VC}_{{\mathbf {B}}_j}$) to ${\mathbf {B}}_j$. On delivery acknowledgement of 9 bit, $\mathbf {BC}$ adds $\mathbf {VC}_{{\mathbf {S}}_i}$ to ${\mathbf {S}}_i$ for which the updated $\mathbf {VC}_{{\mathbf {S}}_i}$ of 32 bits is sent to ${\mathbf {S}}_i$. The total communication cost becomes 73 bits.

The communication cost of the proposed scheme is 640 bits per item.

### Simulation study for QoS validation

**Fig. 7:**
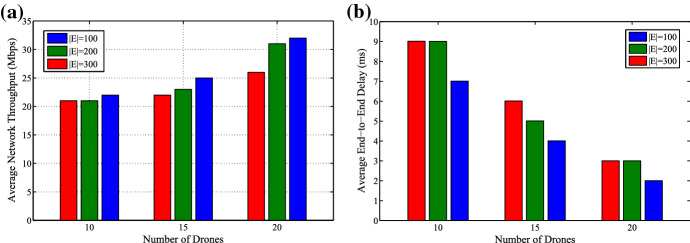
**a** Average network throughput, and **b** Average end-to-end delay

To understand the performance of the proposed scheme, the validation is performed through a simulation study performed using Network Simulator (NS-2). The length of RTS, CTS and ACK packets considered for the evaluations is 170, 120, and 120 bits, respectively. The physical layer header and default packet lengths are same as 802.11b (pause time 2s). The performance results are evaluated on the basis of network throughput and end-to-end delay. To guarantee the QoS satisfaction, a maximum resource utilization must be achieved with an increase in the average transmission rate. The network throughput increases when the achieved utilization rate nears the permissible rate. However, the average network throughput decreases when we increase the number of users. The proposed scheme aims to handle the above challenge by sustaining the average network throughput to its best, even when the number of drones are increased. Fig. 7a shows the variation of the average network throughput with respect to an increase in the number of drones (10, 15, 20) and the number of users (100, 200, 300) requesting delivery.

The end-to-end transmission delay is dependant on many factors and it is not possible to control all these factors. But, the major contributors to the overall delay can be considered as processing and queuing tasks. If both of these tasks are controlled effectively, then it is possible to reduce the overall end-to-end delay. The proposed approach provides a very simple processing model supported by a multi-level queuing scheme for drone scheduling and product delivery. The proposed congestion and collision-aware transmission scheme helps to reduce the overall end-to-end delay in the network. Fig. 7b shows the variation of average delay with respect to an increase in the number of drones (10, 15, 20) and the number of users (100, 200, 300). It depicts that the delay increases with an increase in the number of users in contrast to the number of drones. If we increase the number of drones and reduce the number of users, then a lower delay is observed.

### Complexity comparison in contrast with existing similar works

Various existing proposals have explored the problems related to drones but most of them have not considered the drone delivery case. Here, the proposed work is compared with the existing works in terms of the complexities of the underlying approaches. The findings are summarized in the Table 2.

**Table 2:** Comparison with similar existing works

	[40]	[41]	[42]	Proposed
Drone indexing	$\theta (k*n)$	–	–	$\theta (n)$
Drone scheduling	O(TP+n)	O($n^3$)	O(TP+n)	O(TP+n)
QoS	–	–	–	O(D*$\tau$)

## Conclusion

The proposed framework leverages the advantages of blockchain and smart contracts resulting into more transparent delivery operations COVID-19 like situations. Here, a derived blockchain is proposed for the part of the model where the normal blockchain will not be very suitable candidate still preserving the heritage of immutability of blockchain. In this framework, a virtual currency-based transactions are presented eliminating the reliance on third parties for payment processing hence eliminating the extra burden in terms of monetary cost. One of the key contribution comes from the drone scheduling algorithm which is backed by multi level queuing model ensuring optimal allocation of drones for various delivery jobs with minimal overhead. Incorporation of the QoS aware drone to drone communication approach has brought forward advantage of meeting the most challenging service levels in such scenarios. The proposed framework have been verified and validated for its reliability and performance in a simulated environment. The results indicate the performance of the system is effective. Further, the model is evaluated theoretically for the communication and computation costs which also favours the model.
